# Implication of genetic-dependent stage in the development of *SCN8A*-associated epilepsy: a case report

**DOI:** 10.3389/fnhum.2026.1775909

**Published:** 2026-05-08

**Authors:** Xuetao He, Dongming Zhang, Rong Gan, Jieling Chen, Zisheng Lin, Weijie Liang, Yuhu Zhang

**Affiliations:** 1 Department of Neurology, Guangdong Neuroscience Institute, Guangdong Provincial People’s Hospital (Guangdong Academy of Medical Sciences), Southern Medical University, Guangzhou, China; 2 Department of Neurology, Institute of Neuroscience, Key Laboratory of Neurogenetics and Channelopathies of Guangdong Province and the Ministry of Education of China, The Second Affiliated Hospital, Guangzhou Medical University, Guangzhou, China

**Keywords:** *SCN8A*, genetic-dependent expression, sodium channel blockers, gain-of-function, cluster seizure

## Abstract

The *SCN8A* gene encodes the voltage-gated sodium channel Na_V_1.6, which is essential for neuronal excitability and action potential propagation. *SCN8A* variants are associated with a broad clinical spectrum, ranging from self-limiting syndromes to developmental and epileptic encephalopathies. Here, we identified a novel *de novo* heterozygous *SCN8A* variant (c.791 T > C/p.Val264Ala) in a 19-year-old female patient. This variant was absent in gnomAD and was predicted to be damaging by multiple in *silico* tools. According to the American College of Medical Genetics and Genomics guidelines, the variant was evaluated as “likely pathogenic.” The patient presented with frequent cluster seizures characterized by early onset, remission in childhood, and recurrence in adolescence. The electroencephalograph revealed multifocal epileptiform discharges. The patient was diagnosed with developmental and epileptic encephalopathy. Treatment with sodium channel blockers (oxcarbazepine and lamotrigine) achieved seizure control, a clinical response that suggests a potential gain-of-function effect of the variant. The brain temporal expression of *SCN8A* gradually increases after birth with a peak during infancy, declines through childhood, and rises significantly in adolescence, explaining the development of the patient’s clinical course. This study contributes to the genotype–phenotype correlation of *SCN8A*-related diseases and highlights the implication of genetic-dependent expression (stage) in clinical assessment.

## 1 Introduction

The *SCN8A* gene (OMIM * 600702), located on chromosome 12q13.13, encodes the voltage-gated sodium channel Na_V_1.6, which is a critical regulator of neuronal excitability and action potential propagation (Meisler et al., 2021). In mice, homozygous knockout of *SCN8A* results in postnatal lethality, whereas heterozygous knockout mice exhibits decreased seizure susceptibility.^1^ Heterozygous knock-in mice exhibits premature death, seizures, electroencephalogram abnormalities, and abnormal behaviors (Jones and Meisler, 2014).

In humans, *SCN8A* variants are associated with a wide spectrum of phenotypes, including benign infantile seizures, behavioral/movement disorders, and severe developmental and epileptic encephalopathies (DEEs) (Gardella and Møller, 2019). The *SCN8A*-DEE have been estimated to be the most common form of *SCN8A* related diseases, accounting for 45–68% of cases (Young et al., 2024). Previouse studies have found that missense variants predominantly leading to gain-of-function (GOF) are associated with *SCN8A*-DEE or benign infantile seizures, whereas destructive variants causing loss-of-function (LOF) are associated with autism spectrum disorder, intellectual disability, or movement disorders often without seizures (Blanchard et al., 2015; Gardella and Møller, 2019). However, the association between the genetic expression pattern of *SCN8A* gene and characteristics of the disease course remains underexplored.

In this study, trio-based whole-exome sequencing (WES) was performed in a patient with DEE, identifying a novel *SCN8A* missense variant with a hypothesized GOF effect based on pharmacological response. The seizure course of the patient was characterized by early onset, remission in childhood, and recurrence in adolescence. Further analysis suggested that the temporal expression of *SCN8A* gradually increases after birth with a peak during infancy, declines through childhood, and rises significantly in adolescence, explaining the characteristics of the patient’s clinical course.

## 2 Materials and methods

### 2.1 Patients

Patients with epilepsy without acquired etiology were recruited from Guangdong Provincial People’s Hospital. Clinical information of presented study was collected, including current and seizure onset age, sex, seizure type and frequency, anti-seizure medications (ASMs), neurological physical assessment, brain magnetic resonance imaging (MRI), and long-term video electroencephalography (EEG).

This study was approved by the Ethics Committee of Guangdong Provincial People’s Hospital (EC no. KY2024-244-01). Informed consent was obtained from all subjects involved in the study.

### 2.2 WES and genetic analysis

Blood samples were collected from the patient and the biological parents (trios), and genomic DNA was extracted with QIAamp DNA Blood Mini kit (Qiagen, Hilden, Germany) following the manufacturer’s protocol. The extracted DNA was fragmented randomly and purified using magnetic particle method. DNA fragments were ligated with adaptors and captured by in-house designed probes kit (Nanodigmbio, Nanjing, China) targeting the whole exome. The DNA libraries after enrichment and purification were sequenced on the NovaSeq 6,000 sequencer according to instructions (Illumina, San Diego, United States) according to the manufacturer’s instructions. An average depth of ≥ 90 times was achieved for known exonic regions and flanking 5 bp sequences, with >98% of target bases covered at ≥ 20 times.

All reads were aligned to the Genome Reference Consortium Human Genome build 37 (GRCh37) by Burrows-Wheeler Alignment (BWA). Local realignment and base quality recalibration of the Burrows-Wheeler aligned reads were then performed using the GATK IndelRealigner and GATK BaseRecalibrator,^2^ respectively. SNVs and small indels were identified by the GATK UnifiedGenotyper. Variants were annotated with Annovar.^3^ We selected and interpreted variants associated with the phenotype according to the American College of Medical Genetics and Genomics (ACMG) guidelines (Richards et al., 2015). In this study, *SCN8A* variants were identified and annotated to NM_014191.4. Sanger sequencing of *SCN8A* variants was performed. No pathogenic or likely pathogenic variants in any other genes previously associated with epilepsy or neurodevelopmental disorders were identified in this patient (Zhang M. W. et al., 2024).

### 2.3 Temporal expression profile of *SCN8A* in the human brain

The human RNA-seq data of multiple brain areas was retrieved from the BrainSpan database.^4^ To interpret the genetic expression pattern of *SCN8A*, the temporal expression curve was modeled using a third-order polynomial regression analysis via least squares fitting, as previously reported (He et al., 2024; Luo et al., 2025b; Luo et al., 2025c; Zhang D. et al., 2024), implemented in GraphPad Prism 9.

## 3 Results

### 3.1 Case description

The proband, a 19-year-old female of nonconsanguineous Chinese descent, presented with early-onset seizures at 7 months following an unremarkable perinatal course. Seizures were initially focal to bilateral tonic–clonic, triggered by fever. No relevant family history of neurological disorders was identified. Her developmental milestones were appropriate for age. Initial interictal EEG recorded paroxysmal, asynchronous sharp-and-slow wave complexes involving the bilateral frontopolar, occipital, and regions. Despite treatment with ASMs, she developed recurrent cluster seizures monthly. Between the ages of 4 and 13 years, seizure frequency spontaneously declined to once every 2–3 years without ASMs treatment. After age 13, recurrent cluster seizures increased significantly, with a frequency of once every 2–3 months. Notably, at age 14, she developed cognitive regression and behavioral disturbances, leading to a diagnosed of DEE. Treatment with levetiracetam and valproate proved ineffective, and by age 17, seizure clusters occurred at a frequency of 6–20 episodes per month.

**Figure 1:**
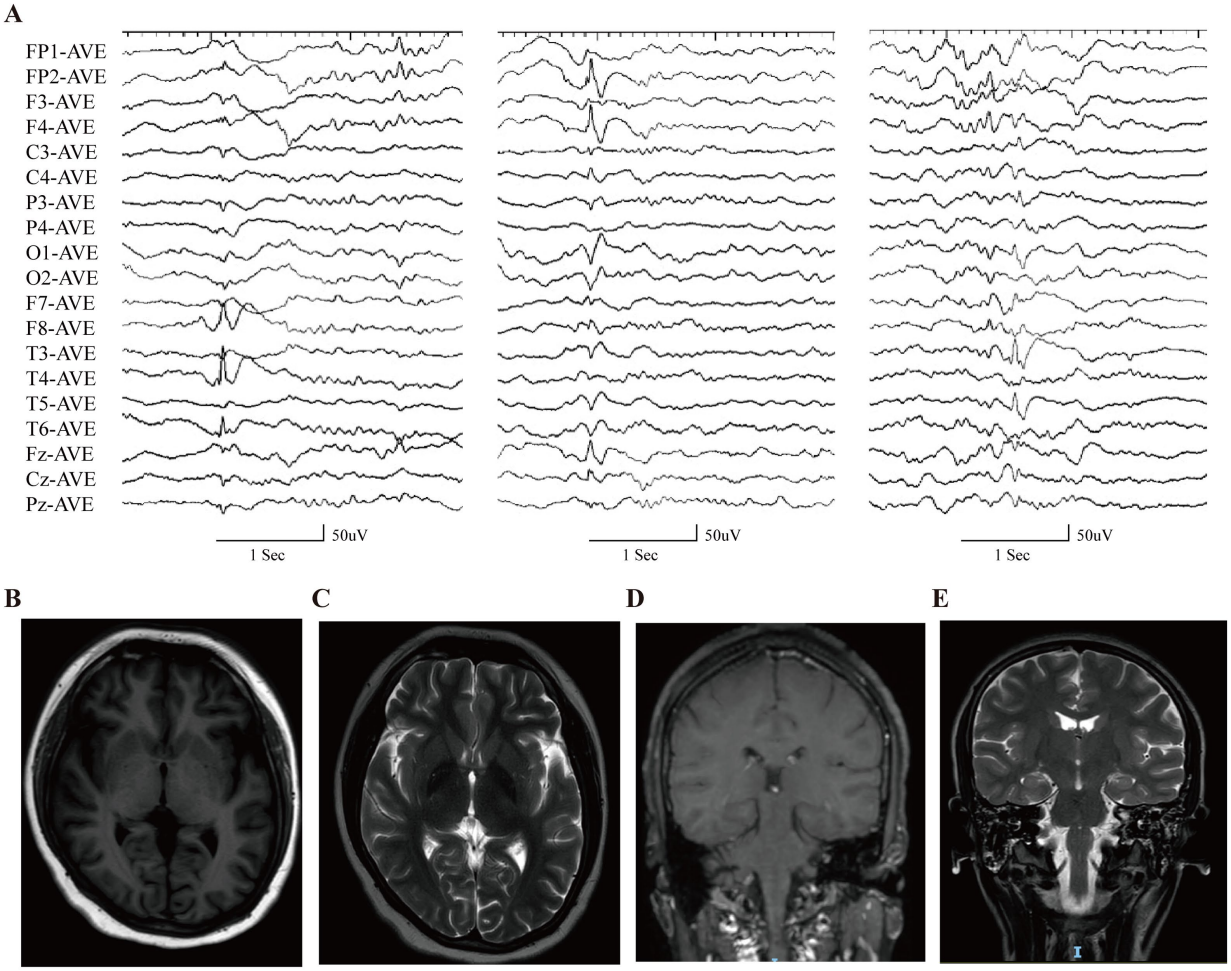
Representative electroencephalography (EEG) and MRI of the case with *SCN8A* variant at 18 years of age. **(A)** Interictal EEG showed multifocal epileptiform discharges in temporal, frontal, and occipital regions. **(B–E)** Brain MRI was unremarkable.

During her initial evaluation at our center at age 18, a neuropsychological assessment indicated severe cognitive impairment, with delayed responsiveness and deficits in comprehension and executive function. Video EEG confirmed multifocal epileptiform discharges in temporal, frontal, and occipital regions (Figure 1A), while brain MRI was unremarkable, showing no evidence of *SCN8A*-associated atrophy or delayed myelination (Figures 1B–E).

**Figure 2:**
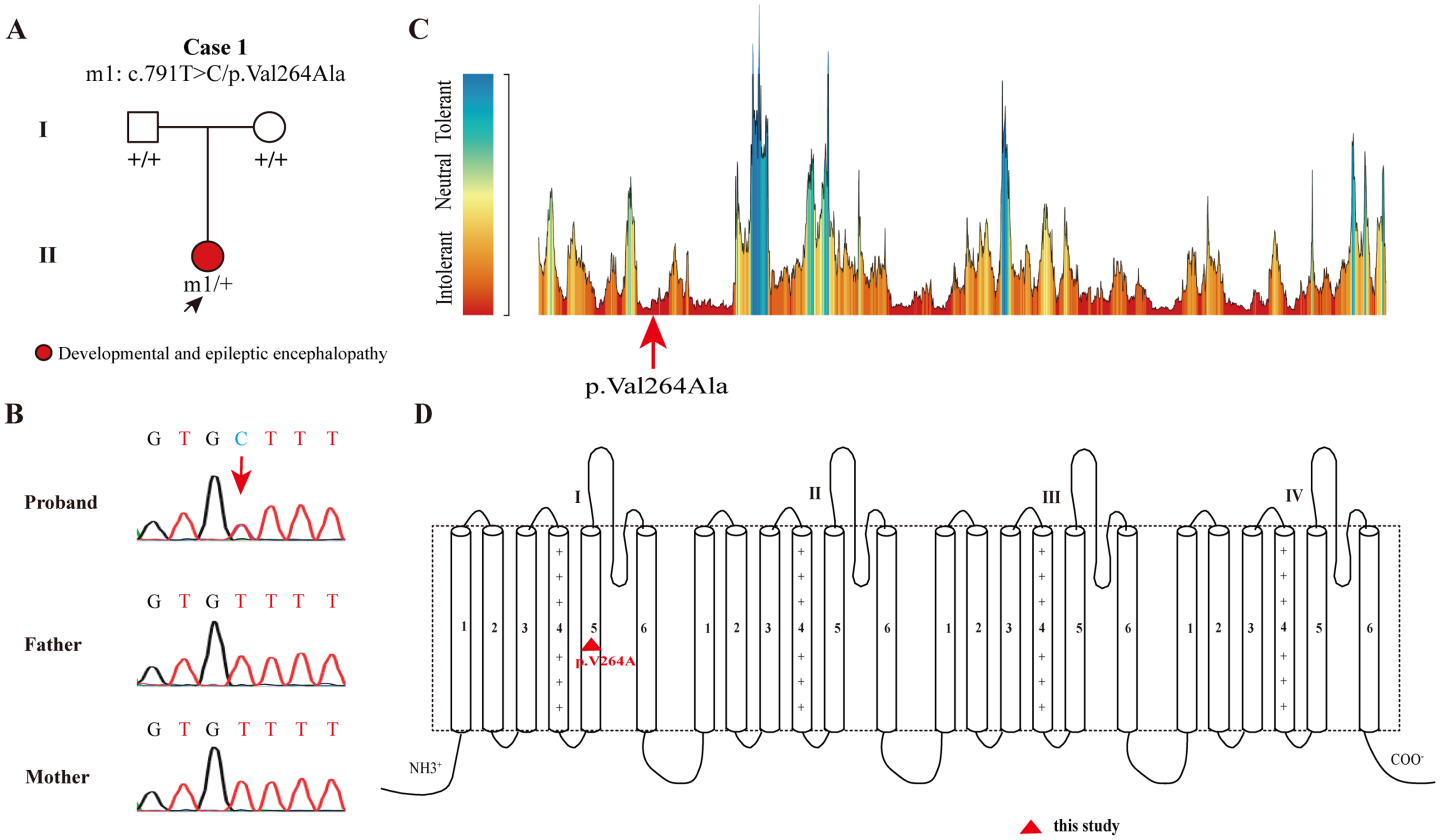
Genetic data and schematic diagram of the voltage-gated sodium channel Na_V_ 1.6 and the localization of *SCN8A* variant. **(A)** Pedigrees of the case with *SCN8A* variant and corresponding phenotypes. **(B)** DNA sequencing chromatograms of the *SCN8A* variant. Arrows indicate the positions of the variants. **(C)** The tolerance of *SCN8A* variant predicted by the MetaDome algorithm. The variant was located in residues that are intolerant of missense variants. **(D)** Schematic diagram of the Na_V_ 1.6 and the localization of the *SCN8A* variant identified in the study.

Trio-based WES identified a novel *de novo SCN8A* missense variant (c.791 T > C/p. Val264Ala), confirmed by Sanger sequencing (Figures 2A,B). This variant was absent in gnomAD and was predicted to be damaging by multiple *in silico* tools, including REVEL, AlphaMissense, SIFT, DANN, and BayesDel. The variant was located in a residues that is intolerant of missense variants based on the MataDome algorithm (Figure 2C).^5^ According to the ACMG guidelines, the variant was evaluated as “likely pathogenic.” The Na_V_1.6 channel includes four homologous domains (I to IV), each containing six transmembrane segments (S1-S6) (Meisler et al., 2016). The variant p.Val264Ala is located in the S5 segment of domain I of the Na_V_ 1.6 channel (Figure 2D).

**Figure 3:**
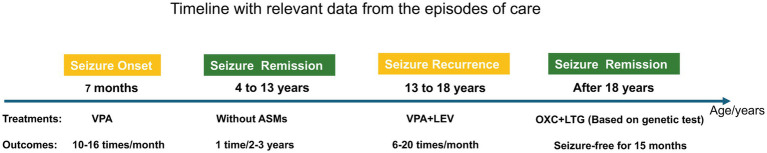
Timeline with relevant data from the episodes of care. ASMs, anti-seizure medications; LEV, levetiracetam; LTG, lamotrigine; OXC, oxcarbazepine; VPA, valproate.

Based on the genetic test results, a combination therapy was initiated at age 18 years, consisting of a reduced dose of levetiracetam and the addition of oxcarbazepine. The dosage of oxcarbazepine was started at 6.5 mg/kg/day in three divided doses and gradually titrated to a maximum of 17 mg/kg/day. Following this adjustment, seizure frequency was decreased with partial improvement in control. Subsequently, lamotrigine was introduced as an add-on therapy at an initial dosage of 0.8 mg/kg/day and titrated up to 1.6 mg/kg/day. This regimen ultimately achieved seizure-free during the follow-up period. Neuropsychological follow-up demonstrated partial cognitive recovery. In accordance with the CARE guideline, the longitudinal timeline, summarizing therapeutic interventions and their respective outcomes, is illustracted in Figure 3. These findings suggest that the functional alteration of the variant p.Val264Ala may be GOF.

### 3.2 Implication of genetic-dependent (expression) stage in *SCN8A*

**Figure 4:**
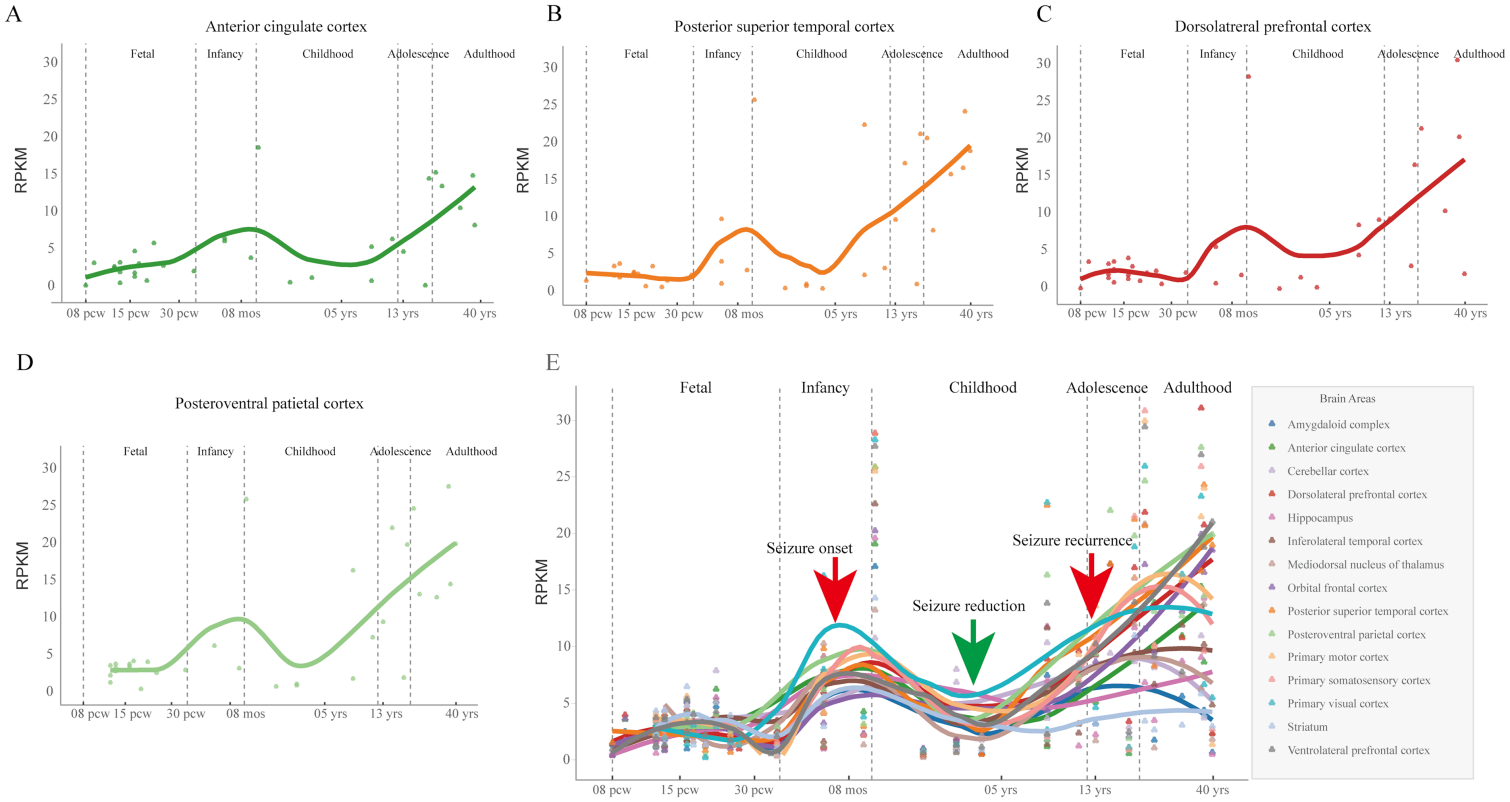
The spatial-temporal expression of *SCN8A* gene. **(A–D)** Temporal expression of *SCN8A* at different developmental stages in the **(A)** anterior cingulate cortex, **(B)** posterior superior temporal cortex, **(C)** dorsolateral prefrontal cortex, and **(D)** posteroventral parietal cortex. **(E)** Aggregated temporal expression of *SCN8A* across various brain regions at different developmental stages. Arrows indicate the clinical course of the patient identified in this study. Curves represent the temporal expression patterns of *SCN8A* in different brain areas. The expression levels were retrieved from the human RNA-seq data in the BrainSpan database. The curve was fitted via the locally weighted scatterplot smoothing (LOWESS) method. RPKM, Reads per kilobase per million mapped reads; pcw, post-conception weeks; mos, months; yrs, years.

Recent studies suggested that the genetic-dependent (expression) stage was associated with the onset age and the outcomes of genetic diseases (He et al., 2023; Luo et al., 2025a; Zhang D. et al., 2024; Zou et al., 2024). In this study, the patient’s epilepsy followed a pattern of early-onset seizures, with remission in childhood and recurrence in adolescence. To analyze the genetic expression pattern of *SCN8A* gene, the human RNA-seq data of multiple brain areas from the BrainSpan database was retrieved. *SCN8A* expression in the brain gradually increases after birth with a peak during infancy, declines throughout childhood, rises significantly in adolescence, and remains elevated in adulthood (Figure 4A–E). The patient’s clinical course was generally consistent with the temporal expression pattern of *SCN8A* in the brain.

## 4 Discussion

This study identified a novel heterozygous *SCN8A* variant (c.791 T > C/p.Val264Ala) in a Chinese patient through trio-based WES. This variant was of *de novo*, had no frequency in gnomAD-all populatuons, and was predicted to be damaging by multiple *in silico* tools. The variant was therefore evaluated as “likely pathogenic,” according to ACMG guidelines. The patient presented with cluster seizures, cognitive regression, and behavioral problems. Based on the genetic test results, oxcarbazepine and lamotrigine were administered, leading to successful seizure control. This therapeutic response suggests a potential GOF effect of the variant c.791 T > C/p.Val264Ala. This study contributes to the genotype–phenotype correlation of *SCN8A*-related diseases. Further study showed that the temporal expression of *SCN8A* gene was generally consistent with the patient’s clinical course.

Pathogenic *SCN8A* variants are associated with a broad spectrum of epilepsy phenotypes, including DEE (45–68%), intermediate *SCN8A*-related epilepsy (8–28%), generalized epilepsy (5%), self-limited infantile epilepsy (2–4%), and neurodevelopmental disorders without epilepsy (3–5%) (Young et al., 2024). *SCN8A*-DEE typically presents with refractory seizures beginning in early infancy, often occurring in clusters (Gardella and Møller, 2019). Here, we report a female patient with seizure onset at 7 months of age and frequent seizure clusters, consistent with *SCN8A*-DEE.

*SCN8A* encodes Na_V_1.6, a channel composed of four transmembrane domains (I to IV), each containing six segments (S1–S6). Four S4 transmembrane segments in Na_V_1.6 act as the voltage sensor. *SCN8A* is widely expressed in both excitatory and inhibitory neurons in the brain and plays an essential role in neuronal excitability (Liu et al., 2021). Na_V_1.6 channels are enriched at the axon initial segment and nodes of Ranvier, where they facilitate action potential initiation and propagation, thereby enhancing neuronal excitability (Blanchard et al., 2015). GOF *SCN8A* variants are associated with *SCN8A*-DEE or self-limited infantile epilepsy, leading to partial or complete hyperactivity of the sodium channel, whereas heterozygous LOF variants cause neurodevelopemetal disorders often without seizures (Blanchard et al., 2015; Gardella and Møller, 2019). The mechanisms of GOF included alterations in electrophysiological properties, such as elevated persistent sodium currents, impaired channel inactivation, and a hyperpolarizing shift in the voltage dependence of activation (Blanchard et al., 2015; Liu et al., 2019). Previous studies have demonstrated that patients with GOF *SCN8A* variants response well to sodium channel blockers, including phenytoin, carbamazepine, oxcarbazepine, and lamotrigine (Gardella and Møller, 2019; Kong et al., 2015; Larsen et al., 2015). In this study, the patient carrying a *de novo SCN8A* missense variant achieved seizure-free with sodium channel blockers (oxcarbazepine and lamotrigine), suggesting a potential GOF effect of the variant.

Recent studies suggest that the genetic-dependent (expression) stage of genes is associated with the age of onset and the clinical outcomes of genetic diseases (He et al., 2023; He et al., 2024; Zou et al., 2024). For example, variants in *SLC2A1* cause glucose transporter type 1 deficiency syndrome (OMIM# 606777), characterized by delayed neurologic development, acquired microcephaly, motor incoordination, spasticity, and refractory seizures (Veneruzzo et al., 2023). The expression of *SLC2A1* in the brain remains high throughout life, with peaks at approximately four and fourteen years of age; this expression pattern provides a mechanistic explanation for the variability in age of seizure onset from early to late childhood (Zhang D. et al., 2024). Similarly, in the present study, the seizure course mirrored the temporal expression of *SCN8A*, which increases postnatally to an infantile peak, declines during childhood, rises substantially in adolescence, and remains high in adulthood. These findings highlight the practical utility of the genetic-dependent (expression) stage in the clinical assessment and management of genetic diseases. The observed correlation between *SCN8A* expression pattern and the seizure course suggests that childhood remission may be associated with developmental down-regulation rather than a permanent resolution of the underlying pathology. Consequently, clinical management should approach the tapering of ASMs with caution and avoid premature drug withdrawal, as the adolescent rise in *SCN8A* expression may represent a potential risk factor for seizure recurrence.

Several limitations should be acknowledged. Functional validation of the p.Val264Ala variant was not performed, and therefore the proposed GOF effect is inferred from clinical evidence. This study represents a single-case design from a single center. Future studies with more extensive data are essential to fully elucidate the association between disease course and genetic-dependent expression pattern.

## Data Availability

The data presented in the study are deposited in the ClinVar database, accession numbers SUB16143583.

## Footnotes

1
https://informatics.jax.org/allele/MGI:1856078

2
https://gatk.broadinstitute.org

3
http://www.openbioinformatics.org/annovar/

4
https://brainspan.org

5
https://stuart.radboudumc.nl/metadome/
